# Large Scale Anthropogenic Reduction of Forest Cover in Last Glacial Maximum Europe

**DOI:** 10.1371/journal.pone.0166726

**Published:** 2016-11-30

**Authors:** Jed O. Kaplan, Mirjam Pfeiffer, Jan C. A. Kolen, Basil A. S. Davis

**Affiliations:** 1 Institute of Earth Surface Dynamics, Géopolis, University of Lausanne, Lausanne, Switzerland; 2 Biodiversity and Climate Research Centre (BiK-F), Frankfurt, Germany; 3 Faculty of Archaeology, Leiden University, RA Leiden, The Netherlands; Clemson University, UNITED STATES

## Abstract

Reconstructions of the vegetation of Europe during the Last Glacial Maximum (LGM) are an enigma. Pollen-based analyses have suggested that Europe was largely covered by steppe and tundra, and forests persisted only in small refugia. Climate-vegetation model simulations on the other hand have consistently suggested that broad areas of Europe would have been suitable for forest, even in the depths of the last glaciation. Here we reconcile models with data by demonstrating that the highly mobile groups of hunter-gatherers that inhabited Europe at the LGM could have substantially reduced forest cover through the ignition of wildfires. Similar to hunter-gatherers of the more recent past, Upper Paleolithic humans were masters of the use of fire, and preferred inhabiting semi-open landscapes to facilitate foraging, hunting and travel. Incorporating human agency into a dynamic vegetation-fire model and simulating forest cover shows that even small increases in wildfire frequency over natural background levels resulted in large changes in the forested area of Europe, in part because trees were already stressed by low atmospheric CO_2_ concentrations and the cold, dry, and highly variable climate. Our results suggest that the impact of humans on the glacial landscape of Europe may be one of the earliest large-scale anthropogenic modifications of the earth system.

## Introduction

**Fig 1:**
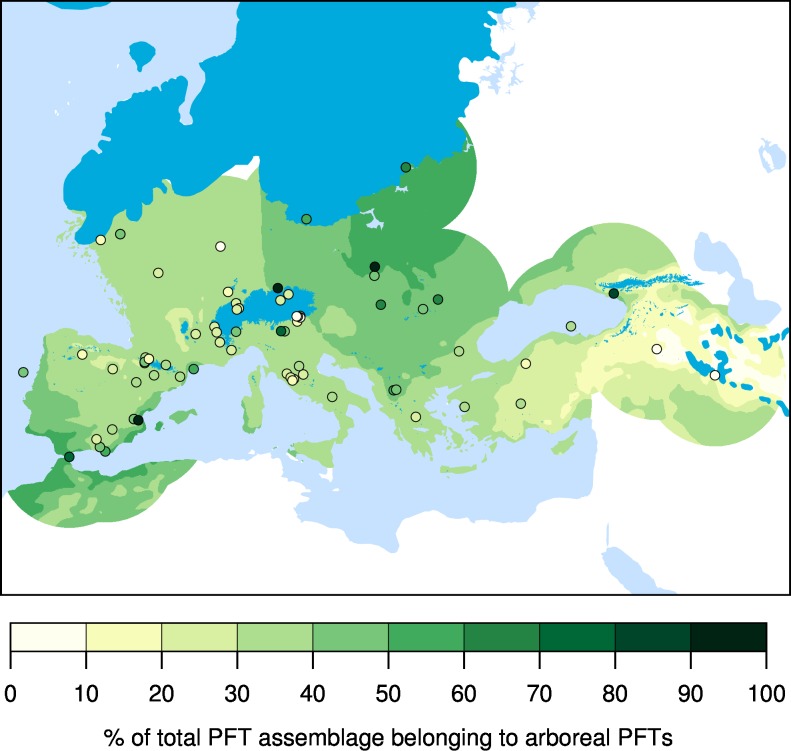
Pollen-based reconstructed tree cover at LGM. Estimated tree cover at LGM (APFT%), calculated as percent sum of all PFT scores belonging to arboreal PFTs. The tree cover surface is based on the 3D interpolation of PFT scores from individual fossil pollen sites (circles). Method following [6]. For data sources see S3 Table.

The land cover of Europe changed substantially since the Last Glacial Maximum (LGM; ca. 21,000 yr cal bp), with important consequences for the development of ecosystems and human society. Reconstructions of vegetation based on pollen and plant macrofossils suggest that most of the non-glaciated parts of Europe was characterized by sparse tree cover (Fig 1), and were largely occupied by steppe and steppe-tundra [1, 2]. Forests may have persisted in restricted areas of southern and eastern Europe, but the location and size of these refugia are debated [3]. In contrast, simulations of the potential land cover of LGM Europe consistently suggest that extensive forests occupied large areas of Europe, particularly north and west of the Alps [4, 5].

While there is little evidence that data-based reconstructions are erroneous, this dichotomy between modeled and reconstructed vegetation cover could be due to inaccuracies in either climate or vegetation models, or due to processes that are largely missing from Earth System Models, such as human agency or animal-vegetation interactions. Climate models may simulate precipitation too great or temperatures too warm to exclude forests from northwestern Europe because of, e.g., an insufficient displacement of the Atlantic storm track under LGM conditions [7]. Alternatively, vegetation models may overestimate the distribution of trees at the LGM by not adequately representing the effects of low atmospheric CO_2_ concentrations, permafrost, soil properties, and disturbance by megaherbivores, on tree growth [8]. Additionally, equilibrium simulations at a specific time-slice may not account for the temporal lag in vegetation dynamics caused by the highly variable climate of the last glacial period [9]. Nevertheless, despite more than two decades of improvements to both climate and vegetation models, the appearance of forests in LGM Europe remains a consistent, robust feature of nearly all simulations [10]. While previous studies have attempted to model the effect of natural fire regimes on LGM vegetation [10, 11], to our knowledge no study so far has included any representation of human agency on LGM vegetation.

**Fig 2:**
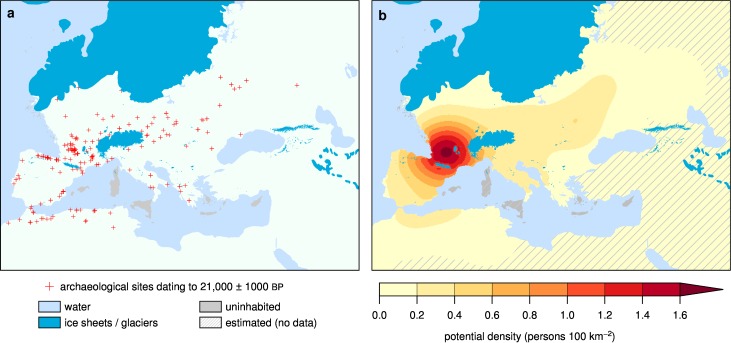
Archaeological evidence for human population in LGM Europe. (A) Distribution of archaeological sites dating to LGM [17, 18], (B) estimated baseline population density using kernel interpolation following [19].

Behaviorally modern humans (*Homo sapiens sapiens*) entered Europe as early as 48 thousand years ago (ka), and by the LGM had completely displaced all other hominin species that previously lived on the continent, including Neanderthals [12]. Archaeological evidence shows that modern humans were present throughout Europe and North Africa at the LGM (Fig 2A). Upper Paleolithic hunter-gatherers were highly mobile foragers with sophisticated toolkits and organized social structures [13]. Fire was a significant part of hominin technology for at least the last 300 ka [14], and, based on historical analogues, we hypothesize that hunter-gatherers at the LGM had mastered the use of fire as a tool for landscape management, for improving hunting and foraging opportunities, and easing visibility and travel [15]. To investigate the potential impact of humans on the landscapes of Europe at the LGM, we ran a coupled human demographics, vegetation, and disturbance model (LPJ-LMfire) [16] driven by simulated LGM climate from eight climate models.

## Results

**Fig 3:**
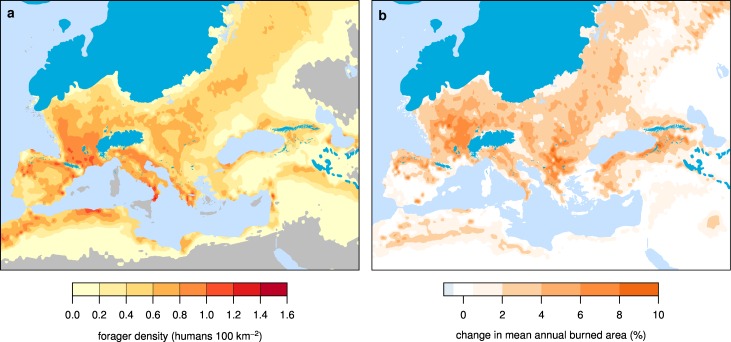
Forager population density and human induced fire at LGM. (A) Forager population density, and (B) the change in mean annual burned area from the LPJ-LMfire ensemble mean scenario.

Modeled hunter-gatherer demographics show that human population was concentrated in southwestern Europe at the LGM, particularly in southwestern France and northeastern Spain, but higher densities of hunter-gatherers were also present in southern Italy and along the northwest coast of Africa (Fig 3A). Simulated hunter-gatherer populations vary dynamically as a result of climate variability and land cover change feedbacks induced by anthropogenic burning, which favors herbaceous vegetation at the expense of forests [16].

**Fig 4:**
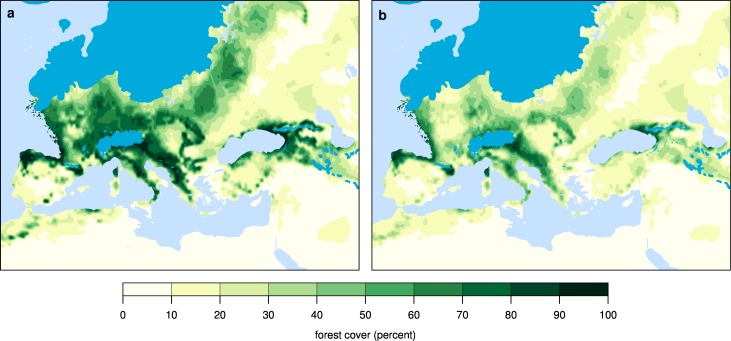
Influence of anthropogenic burning on tree cover in LGM Europe. (A) Tree cover simulated without and (B) with human burning. Results are from the LPJ-LMfire ensemble mean scenario.

The simulated rate of anthropogenic burning is proportional to humans’ ability to maintain a semi-open landscape, i.e., a heterogeneous mix of trees and herbaceous vegetation [16]. Comparing simulations of burned area in LGM Europe with and without anthropogenic fire (Fig 3B) reveals that hunter-gatherer ignitions result in a modest absolute increase in mean annual burned area, reaching a maximum of less than 10% in limited areas north and west of the Alps, in the Balkans, and along the southern coast of the Black Sea. In general, anthropogenic fire leads locally to a 2–4% increase in burned area above natural background levels. This modest absolute increase in burning results, however, in large reductions in forest cover (Fig 4; S1 and S2 Figs), particularly north and west of the Alps, in the eastern Balkans, and in the central part of eastern Europe between the Fennoscandian Ice Sheet and the Black Sea.

Simulated burned area varies considerably depending on the climate model used to drive LPJ-LMfire, ranging from 0.79–1.32 x 10^6^ km^2^ in experiments without human fire, and from 1.01–1.55 x 10^6^ km^2^ in simulations with anthropogenic ignitions (S1 Table). In the multi-model ensemble mean simulation, total mean-annual burned area increases by ca. 20% as a result of human activities. Simulated forest cover showed similar variability across climate model scenarios ranging from 3.13–4.93 x 10^6^ km^2^ in experiments without human fire and from 1.81–4.07 x 10^6^ km^2^ in simulations with anthropogenic ignitions (S2 Table). The multi-model ensemble mean reduction in forest cover is nearly 30%, suggesting that even though the absolute anthropogenic increase in burned area is relatively modest, it leads to a disproportionate reduction in forest cover.

Trees in Europe would have been stressed at the LGM as a result of low atmospheric CO_2_ concentrations and cold, variable climate [9, 20]. In our simulations, the additional disturbance caused by anthropogenic burning leads to increased tree mortality and suppressed regeneration–tree seedlings and saplings are much more sensitive to fire than mature individuals–resulting in more open landscapes over much of the continent. Our results suggest that the discrepancy between the high degree of landscape openness indicated by pollen-based vegetation reconstructions (Fig 1) [1, 2] and the high fractions of tree cover simulated in previous vegetation model simulations [4, 8] may be reconciled by accounting for fire caused by hunter-gatherers, both intentional and accidental.

**Fig 5:**
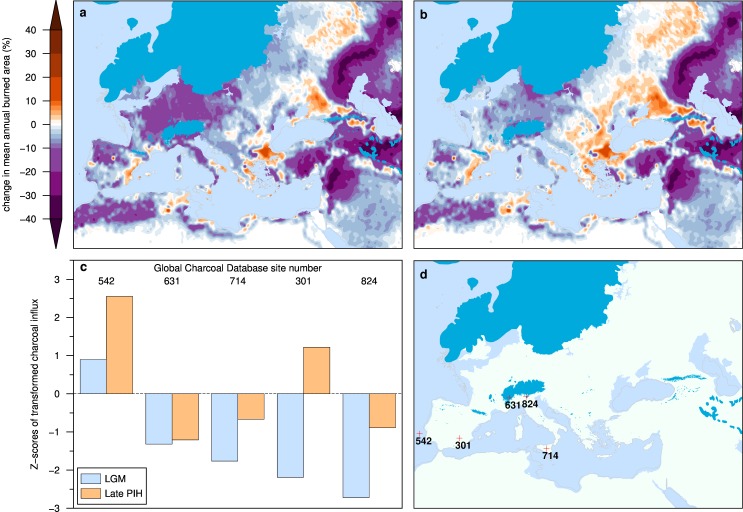
Change in burned area fraction between LGM and the Preindustrial Holocene (PIH). Difference in mean annual burned area fraction between LGM and the late PIH (AD 1770) in (A) the LPJ ensemble mean simulation without human burning, and in (B) the LPJ ensemble mean scenario including human burning. Panel (C) shows relative charcoal accumulation at the LGM and late PIH (AD 1650±200 yr) at the five Global Charcoal Database sites (D) containing lake and marine sediment microcharcoal dating to LGM [23]. The Z-scores of transformed charcoal influx are calculated relative to the preindustrial Holocene base period (0.2–12 ka) following [21]. At all charcoal sites the charcoal influx is smaller in samples dated to LGM compared to the PIH. The gray outline is the LGM coastline.

To evaluate our simulations in the context of independent estimates of changes in fire activity, we compared a model run for the late preindustrial Holocene (PIH, ca. AD 1770) to our simulations for LGM and to records of sedimentary charcoal (Fig 5, S3 Fig). Sedimentary archives from Europe contain consistently less charcoal dated to LGM compared to the PIH [21]. While a direct quantitative comparison between simulated burned area and charcoal abundance is impossible, our model experiments qualitatively agree with the charcoal records, showing substantially lower burned area at the LGM, even in simulations with anthropogenic burning at the LGM. A few areas show more fire at the LGM compared to PIH, e.g., along the eastern coast of Iberia, and in Thrace and Ciscaucasia. In these regions, increases in burned area are apparent in both simulations with and without anthropogenic fire, suggesting that the effect is mainly related to climate change between LGM and PIH, with a small amplification caused by human burning. Comparison of ice-free land pixels common to both simulation periods yields a mean increase in burned area between LGM and PIH of ca. 60% (from 1.09 x 10^6^ km^2^ to 1.74 x 10^6^ km^2^). This increase in burned area is about triple the difference between LGM simulations with and without anthropogenic ignitions, and is due in part (ca. 19%) to the widespread burning of agricultural fields and pastures that was common practice before the industrialization of agriculture in 20^th^ century Europe [22].

## Discussion

The pollen-based tree cover reconstruction presented in Fig 1 suggests that the landscapes of Europe were not completely treeless at LGM. While all of the pollen samples in the dataset we prepared for this analysis are securely dated to the LGM, the data are very sparse in many parts of our study area, including Europe north and west of the Alps, southwestern Iberia and North Africa, and in the northwestern Balkans. This leaves us unable to resolve microenvironments that could have been refugia for trees [24], which could lead to an underestimation of the tree cover in some regions. In contrast, the pollen samples may not be completely representative of the larger landscape and could therefore also over-represent tree cover in places. Nevertheless, our data synthesis represents the most comprehensive collection of LGM pollen samples collected to-date.

While very little direct evidence is available with which to contrast vegetation and fire under full glacial conditions with and without the presence of modern humans, pollen and charcoal records dating to the LGM provide an indication of environments of glacial Europe. A recent study on microcharcoal recovered from marine sediments offshore of Western Europe [25] suggested that no change in the frequency or magnitude of biomass burning was detectable following the arrival of modern humans in Europe around 40 ka. However, one of the two cores (MD95-2042) analyzed in that study was recovered off of southwestern Iberia, where our modeling results also indicate an insignificant change in both fire frequency and vegetation cover under human influence. The second marine core (MD04-2845), from farther offshore western France, does not have any samples that cover the LGM period that is the subject of this study. Nevertheless, we expect that any glacial-age anthropogenic burning signal would be difficult to detect at a marine location for the following reasons: 1) in our simulations, the increase in fire frequency caused by humans is modest, 2) the sedimentation process at deep-water marine core sites is highly uncertain–rapid fluctuations in sea level and ocean circulation during the last glacial period could mask an onshore trend [26, 27]– 3) the large magnitude of climate fluctuations between stadials and interstadials would overshadow human-induced changes when observing a period covering several cycles, and 4) the marine sediment records integrate over very large areas, e.g., mountains and lowlands, while the potential influence of humans on the landscape might have been more localized. A more illustrative test of our hypothesis would be to contrast paleoenvironmental reconstructions of full-glacial conditions recovered at a terrestrial site. Pollen and charcoal records from the Balkans suggest that the Penultimate Glacial Maximum (ca. 130 ka), a time before modern humans were present in Europe, was characterized by less fire and greater forest cover as compared to LGM [28].

The case for large-scale human influence on the vegetation cover of LGM Europe thus rests on four arguments: 1) the LGM climate was not adverse enough to exclude woody vegetation from most of Europe, 2) there is little evidence of a high frequency of lightning-ignited wildfire, 3) Pleistocene megafauna did not preferentially browse woody vegetation and were not present in sufficient density at the LGM to substantially affect the vegetation and 4) the evidence of highly-mobile populations of modern human throughout LGM Europe and their inferred behavior suggests that anthropogenic fire, both intentional and accidental, may have been a unique feature of the LGM as compared to earlier periods.

Although climate change was an important driver of landscape change during the LGM, paleoclimate alone cannot not provide sufficient explanation for the large-scale open nature of LGM landscapes. While the openness of the LGM landscape itself may be debated–our tree-cover reconstruction (Fig 1) and a number of previous studies [3, 24, 29–31] confirm the notion that Europe at the LGM was probably not a homogenous, vast open steppe, but rather characterized by a patchy mosaic of forest and non-forest vegetation–no GCM simulation results in a modeled vegetation of Europe that is even remotely treeless (S1 Fig). The LGM simulation of some climate models does lead to the simulation of a large reduction in tree cover close to the Fennoscandian Ice Sheet, but the dense tree cover simulated in parts of Europe by the vegetation model without human influence is not consistent with any of the paleoecological reconstructions. The high-frequency, high-magnitude climate variability that characterized earlier parts of the last glacial, e.g., MIS 3, may have had a significant effect on vegetation [9], but at the LGM the major climate fluctuations subsided for several thousand years [32]. These results indicate that paleoclimate conditions in Europe at the LGM were favorable enough to support extensive woody vegetation.

It is unlikely that the treeless landscapes of the LGM were an outcome of increasing frequency of lightning-ignited wildfires. A very high frequency of natural wildfire at the LGM would appear to be at odds with both reconstructions of low lightning frequency [33] and charcoal records [25].

It is equally unlikely that the reduction in tree cover during the LGM was a result of megafauna feeding behavior. While the Wooly Mammoth is the iconic animal of the late Pleistocene, it has been argued both that the open vegetation of LGM Eurasia was not productive enough to support very large densities of grazing and browsing animals [34], and that woody vegetation did not comprise a significant part of the Mammoth diet [35], at least in northeastern Asia. Furthermore, observations and modeling studies in landscapes with much greater primary productivity than LGM Europe, e.g., African savannas, suggest that even high densities of wild animals have limited impact on large-scale vegetation patterns [36].

Archaeological evidence demonstrates that humans were present throughout Europe at the LGM [18, 19, 37] and their inferred behavior suggests that these people were well aware of using fire as a tool for improving mobility and opportunities for hunting and gathering [25].

It is therefore important to consider the changes in human behavior that occurred towards the end of the last glacial cycle. Immediately before the LGM, Mid-Upper Paleolithic hunter-gatherers in Europe employed advanced pyro-technologies for a wide range of purposes, and constructed high-investment and durable on-site facilities, such as hearths that were reused over multiple phases of occupation [38–40]. Under the harsh environmental conditions of the LGM, food, fuel, and natural shelter became more sparsely distributed. As a response, hunter-gatherers seem to have increased their mobility at various spatial scales [41, 42] and established large-scale alliance and social safety networks [43] to cope with the climatic degradation and make most effective use of what resources were available. The distances over which lithic materials and ornaments [44], stylistic repertoires, and even genes were exchanged and/or transported increased significantly after ca. 30 ka [41], constituting wide-ranging networks that covered increasingly large territories. Compared to earlier periods of the Last Glacial, i.e., MIS 3, hunter-gatherers of LGM Europe invested less time, matter and energy in their bivouacs and although fire was used frequently, well-built hearths and elaborate dwellings, and other durable facilities such as burials [45], are scarce, less evident in the archaeological record, or remain disputed [41, 46, 47].

Archaeological evidence thus suggests that European hunter-gatherers were highly mobile and well experienced in the use of fire as part of their toolkit by the LGM. Fire must have been of vital importance for human survival in the cold landscapes of LGM Europe. Indeed, there is widespread evidence for fire in LGM Europe, both at archaeological sites and in off-site situations. Open-air archaeological sites consistently show evidence of fire use, even where wood for fuel must have been extremely scarce, including small and unprotected fireplaces, thin ash lenses, widespread micro-charcoal remains, charred bone and burned flint artifacts [48–51]. Numerous loess profiles dated to the LGM are characterized by ash layers containing micro-charcoal remains, including locations in the Carpathian Basin [49], Ukraine and Central Siberia [50], and as far east as the northern Chinese Loess Plateau [52]. Human use of fire in unprotected and unmaintained settings in the semi-open, semi-arid landscapes of glacial Europe would have increased the risk of wildfire. Accidental ignitions, in addition to targeted burning to facilitate hunting and mobility, likely led to a modest but ecologically important increase in fire frequency compared to the natural background.

Evidence from material culture does indicate that Paleolithic hunter-gatherers in much of Eurasia were big-game hunters and relied on the exploitation of large herbivores for an important part of their diet [47]. While megafauna densities at LGM are extremely poorly constrained [53], we cannot completely exclude that the vegetation of LGM Europe was at least partly the result of an interplay between anthropogenic fire and megafauna influences on the vegetation. Under certain conditions, very high densities of large herbivores have been shown to have a strong effect on sub-Arctic vegetation [53]. Future model simulations should therefore aim to better quantify the potential effect of megafauna alongside human agency in influencing the landscapes of LGM Eurasia, and contrasting this with, e.g., North America, where humans were not present at LGM.

Both the influence of Paleolithic hunter-gatherers and megafauna on the large scale landscapes of Eurasia may have been phenomena particular to the LGM. In the late Pleistocene and early Holocene, rising atmospheric CO_2_ concentrations, followed by intense warming and increases in humidity led to the rapid development of closed forests in most of northern and western Europe (e.g., [54, 55]). These forests would have been much more difficult for humans to burn, and indeed there is little evidence for anthropogenic burning in Europe in the early Holocene [56], while at the same time a large part of the Pleistocene megafauna went extinct [57]. While the influence of both humans and large herbivores on European forests in the Holocene is debated [58], in most northern and western European forests, the primary agent of forest disturbance during the Holocene was, and continues to be, wind-throw [59, 60], excepting anthropogenic deforestation that became widely important starting in the Bronze and Iron Ages (4–3 ka) [61].

The low atmospheric CO_2_ concentrations, and cold, variable climate of the LGM stressed woody vegetation, but without additional disturbance most model simulations predict a largely forested Europe. While the feeding behavior of Pleistocene megafauna has often been cited as a primary cause of this additional disturbance, recent analyses of diet and potential animal density suggest that the impact of wild animals would have been too small on their own to create large, open landscapes. On the other hand, our model results suggest that hunter-gatherers, through the occasional ignition of fire, caused substantial reductions in tree cover and the promotion of heterogeneous, low-density woodlands; landscapes that are consistent with pollen-based tree cover reconstructions. This anthropogenically induced reduction in forest cover may be one of the earliest examples of large-scale human modification of landscapes and emphasizes the need to take human action into account when reconstructing land cover of the Late Glacial and early Holocene.

## Methods

### Summary

We used the LPJ-LMfire dynamic global vegetation model [16] to quantify the potential effects of human-caused fire on European forest cover at the LGM. The model contains an explicit representation of the use of fire by hunter-gatherers and a new module to simulate hunter-gatherer demographics. We performed model experiments using simulated paleoclimate from a range of climate models that participated in a common experiment, both including and excluding anthropogenic ignitions of wildfire for each climate scenario. For comparison with reconstructions of past fire activity based on charcoal recovered from sediment archives, we also ran a simulation representing the climatic, geographic, and demographic conditions in the late preindustrial Holocene (ca. AD 1770).

### Paleoclimate scenarios

To simulate fire frequency, burned area, tree cover and primary productivity, we forced LPJ-LMfire with a paleoclimate scenario based on monthly mean paleoclimate anomalies (temperature, precipitation, cloud cover, windspeed) from eight global climate models (GCMs) that had performed the PMIP3/CMIP5 LGM experiment [62], which prescribes boundary conditions for paleotopography, ice cover, orbital forcing, and greenhouse gas concentrations. For GCMs providing several ensemble members per time slice, we used the respective GCM’s ensemble mean for our simulations. We estimated the number of wet days per month and grid cell as a function of total monthly precipitation based on a geographically localized linear regression for present-day climate (P. J. Bartlein, pers. comm., 2012). Anomalies for each of the paleoclimate variables listed above were created by subtracting preindustrial control run values from the LGM values at the native spatial resolution of each GCM. The resulting anomalies were bi-linearly interpolated to a 0.5 degree geographic grid and added to the standard climatological forcing used for LPJ-LMfire and described in [16].

We were not able to estimate a paleoclimate anomaly for lightning strikes because the necessary variables [33] were not archived in most of the PMIP3 GCM output. We therefore used the late-20^th^ century lightning strike rate from the modern baseline dataset [16] in the LGM simulations. While this represents an uncertainty in our model simulations, it is consistent with the insignificant LGM-to-PIH changes in lightning strikes observed in the temperate and boreal latitudes in model simulations by [33].

Interannual climate variability was imposed by adding the corresponding paleoclimate scenario to a detrended interannual climate timeseries, which was created by assembling randomly selected 30-year-blocks of climate from the detrended 20^th^ Century Reanalysis dataset as described in [16].

For each of the resulting LPJ-LMfire climate data input sets we performed one baseline simulation allowing only natural, i.e., lightning-caused ignitions, and a second simulation that included anthropogenic ignitions along with lightning. For the preindustrial simulation we included anthropogenic burning by farmers and pastoralists following the scheme described in [16] and using population and land use data from [63]. The procedure we used to simulate hunter-gatherer population density at the LGM is described below.

### LGM land surface conditions

For the LPJ-LMfire simulations we developed a high-resolution (0.5-degree) reconstruction of topography, dry land area, and soils at the LGM. To determine LGM topography, we added an LGM paleotopographic anomaly surface to a modern high-resolution digital elevation model. The paleotopographic anomaly was calculated by differencing the sub-ice topography between LGM and present-day using results of the ICE-5G ice sheet model [64]. We bilinearly interpolated the topographic anomaly to 1 arc-minute resolution and added this field to the ETOPO1 [65] present-day digital elevation model. We then applied a flood-fill algorithm to determine areas not covered by water. Sea level in the oceans was set at the zero contour in the paleotopography dataset (approximately 120 m below present day sea level [64]). We specified levels of the inland Eurasian seas and isolated basins as follows (m above present sea level): Black Sea, −151 m [66]; Caspian Sea, −120 m [67]; Aral Sea, 31 m [68]; Sea of Marmara, −85 m [69]; Gulf of Corinth, −70 m [70]. The Red Sea and Gulf of Aqaba were assumed to be connected to the global oceans at the LGM [71]. We further masked the resulting land-only topography dataset with the outlines of the LGM continental and mountain glaciers from [72] with modifications for the British-Irish Ice Sheet from [73], rasterized at 1 arc-minute resolution. In the Alpine region, glaciers covered the surface of nearly the entire range (ca. 300,000 km^2^), compared to about 2,270 km^2^ in AD 2000. We then calculated the slope of each gridcell following [74]. Finally, we filtered the 1 arc-minute ice-free land elevation and slope grids to 0.5 degree resolution, resulting in separate files for ice-free land fraction, and median elevation and slope. When directly comparing LGM scenarios to the PIH simulation, only areas that were land during both time windows were considered for evaluation. Soil physical properties were prescribed from the Harmonized World Soil Database (HWSD) [16, 75]. We resampled the soil data from the native 30 arc-second resolution of the HWSD to a 0.5 degree grid using a modal filter, and for land exposed at the LGM due to the lowered sea level, we extrapolated from the nearest non-missing neighbor in the HWSD.

### Simulating hunter-gatherer population density

We simulated hunter-gatherer population dynamics as a function of the landscape carrying capacity and a simple demographic model based on the Verhulst equation [76]. Carrying capacity was estimated as the combination of a logistic function based on vegetation net primary productivity (NPP) and a Gaussian function based on tree cover. Following [77], optimum carrying capacity was assumed to be achieved with intermediate levels of both NPP and tree cover. We estimated that maximum NPP-related carrying capacity was achieved in the region with the highest reconstructed population densities in LGM Europe, in southwest France [19]. Extracting NPP for southwest France from a baseline multi-model ensemble mean LPJ-LMfire LGM simulation without humans resulted in a typical value of approximately 500 g m^-2^; we used this threshold as the point at which the logistic function reaches its maximum. The logistic function for NPP-based carrying capacity was therefore

$$C_{NPP}=\frac{1}{{\left(1+0.15e^{-0.015NPP}\right)}^{50}}$$

where NPP is annual vegetation net primary productivity and C_NPP_ is a scalar value from 0–1 with a lower limit at NPP = 0 and a maximum at NPP ≈ 500 g m^-2^. The Gaussian function for the optimum tree-cover influenced carrying capacity is

$$C_{tree}=e^{\frac{-{\left(T-0.5\right)}^{2}}{0.230}}$$

where T is the fractional cover of trees in the gridcell and C_tree_ is a scalar suitability index, with a maximum value of 1 at T = 0.5 and minima of about 0.1 at T = 0 and 1. Maximum real carrying capacity, where both C_NPP_ and C_tree_ = 1, was fixed to 1.646 persons per 100 km^2^ following [19].

To initialize human populations at the beginning of the simulation, we created a baseline map of reconstructed hunter-gatherer population densities in Europe. This reconstruction (Fig 2B) is based on a dataset of 335 archaeological sites (Fig 2A) securely ^14^C dated to the LGM (21’000 ± 1000 Cal. BP) from [17, 18]. We interpolated between archaeological sites using a kernel function following [19] to create a density map suitable for input into the coupled human-environment model.

The baseline population growth rate for hunter-gatherers was prescribed at 8x10^-5^ yr^-1^ following [78] and using the Verhulst equation, the growth rate slowed as population neared gridcell-level carrying capacity. Carrying capacity was recalculated annually in the model simulations based on the LPJ-LMfire annual simulations of NPP and tree cover fraction. If total population of a gridcell was above the annual carrying capacity, population was reduced to that year’s carrying capacity. Emigration to neighboring gridcells was not considered. If the total number of people in a gridcell fell below a minimum group size of 10, the gridcell was considered to be uninhabited and population was set to zero. In subsequent years, if carrying capacity was greater than the minimum group size, the gridcell was reinitialized to an initial population density corresponding to 10 people living in the gridcell.

### Model simulation protocol

The model was run on a 0.5° geographic grid (approximately 50 km). We ran the model in a series of experiments with and without human presence, bringing the model to equilibrium by performing a 1020 year simulation under LGM climate conditions following the protocol described in [16]. For evaluation and presentation of the model output, we averaged the last 150 years of the simulation, when we assume vegetation and human population densities to be in equilibrium with climate. We performed one LPJ-LMfire run for each of the LGM GCM scenarios, and one simulation using the mean of the climate anomalies taken across all of the GCMs (the “GCM mean” scenario). Finally, we averaged the LPJ-LMfire output across each of the eight individual GCM simulations, but not including the GCM mean; this output is called the “LPJ mean” result. We also performed a simulation for late preindustrial time using a population and land use scenario, and climate data detailed in [16].

### Pollen-based tree cover reconstruction

To estimate the tree cover of Europe at LGM (Fig 1) we assembled a database of pollen records dated to 21,000±1000 cal. BP based on the European Pollen Database and a number of other published sources. We classified pollen spectra into plant functional types (PFTs) and aggregated these to arboreal versus non-arboreal percentages following [6]. The tree cover reconstruction presented in Fig 1 is based on 71 pollen sites from across Europe, representing a significant improvement in both spatial coverage and dating quality compared to previous synthesis studies. For instance, the BIOME6000 LGM dataset for Europe [79] was based on only 18 pollen sites, of which 10 were classified as ‘poorly dated’ under the COHMAP dating classification scheme. In contrast, we have excluded all sites with the ‘poorly dated’ classification in our synthesis. The BIOME6000 analysis had previously suggested that the most of Europe was covered in steppe, but with increased site coverage, and a more sensitive measure of forest cover based on arboreal pollen percentages, we find a much more variable picture. Our reconstruction shows areas of potentially quite dense tree cover in central and south-western areas of Europe, with which the model simulations agree. In most of the rest of Europe, we reconstruct a mixture of steppe and open woodland. Our analysis further shows some sites close to the ice sheet edge with evidence of relatively high levels of arboreal taxa, suggesting pockets of woodland were able to survive in sheltered areas [80]. The possibility that the presence of arboreal taxa is the result of long distance transport or reworking is considered unlikely given the relatively high levels of arboreal pollen involved, and direct supporting evidence based on macrofossils [3, 81].

## Supporting Information

S1 Fig Tree cover in simulations with and without human burning. Tree cover fraction simulated by LPJ-LMfire using each of the eight GCM LGM climate simulations without human burning (**left panels**) and with human burning (**right panels**). LPJ-LMfire results are also shown for a simulation using the multi-model ensemble mean climate (**GCM mean**), and the mean of all of the individual LPJ-LMfire simulations (**LPJ mean**).
(PDF)

S2 Fig Simulated forager population density and associated reduction in tree cover. The reduction in tree cover between a simulation with and without human burning (**left panels**) and the associated simulated forager population density (**right panels**). Results are shown for LPJ-LMfire runs driven by each of the eight GCM climate simulations, and the multi-model ensemble means.
(PDF)

S3 Fig Change in burned area fraction between LGM and the Preindustrial Holocene (PIH). Difference in mean annual burned area fraction for each of the individual GCM LGM climate scenarios compared to a PIH simulation. **Left panels**, scenarios without human burning at LGM, **right panels**, with human burning.
(PDF)

S1 Table 150-year mean burned area for the map area covered by Fig 1 for the eight different GCM climate input scenarios, and one scenario using the mean climatology of the 8 different scenarios. Values in parentheses are the 1-σ temporal variability of the individual GCM scenarios, and the 1-σ variability across scenarios for the LPJ mean.
(PDF)

S2 Table 150-year mean tree cover for the map area covered by Fig 2 for the eight different GCM climate input scenarios, and one scenario using the mean climatology of the 8 different scenarios. Values in parentheses are the 1-σ temporal variability of the individual GCM scenarios, and the 1-σ variability across scenarios for the LPJ mean.
(PDF)

S3 Table List of samples used to create the pollen-based reconstruction of tree cover at LGM presented in Fig 1. (A) Complete metadata for entities contributed to the European Pollen Database (EPD) and Pangaea data library may be found at their respective web sites (EPD, Pangaea). (B) Legend for the dating code column in S3A Table.
(PDF)
