# Supplementary material for: Large Scale Anthropogenic Reduction of Forest Cover in Last Glacial Maximum Europe
Source: PLoS One. 2016 Nov 30;11(11):e0166726. doi: 10.1371/journal.pone.0166726 (PMC5130213; doi:10.1371/journal.pone.0166726)
Supplement: S1 Fig — Tree cover fraction simulated by LPJ-LMfire using each of the eight GCM LGM climate simulations without human burning (left panels) and with human burning (right panels). LPJ-LMfire results are also shown for a simulation using the multi-model ensemble mean climate (GCM mean), and the mean of all of the individual LPJ-LMfire simulations (LPJ mean). (PDF) [file pone.0166726.s001.pdf]

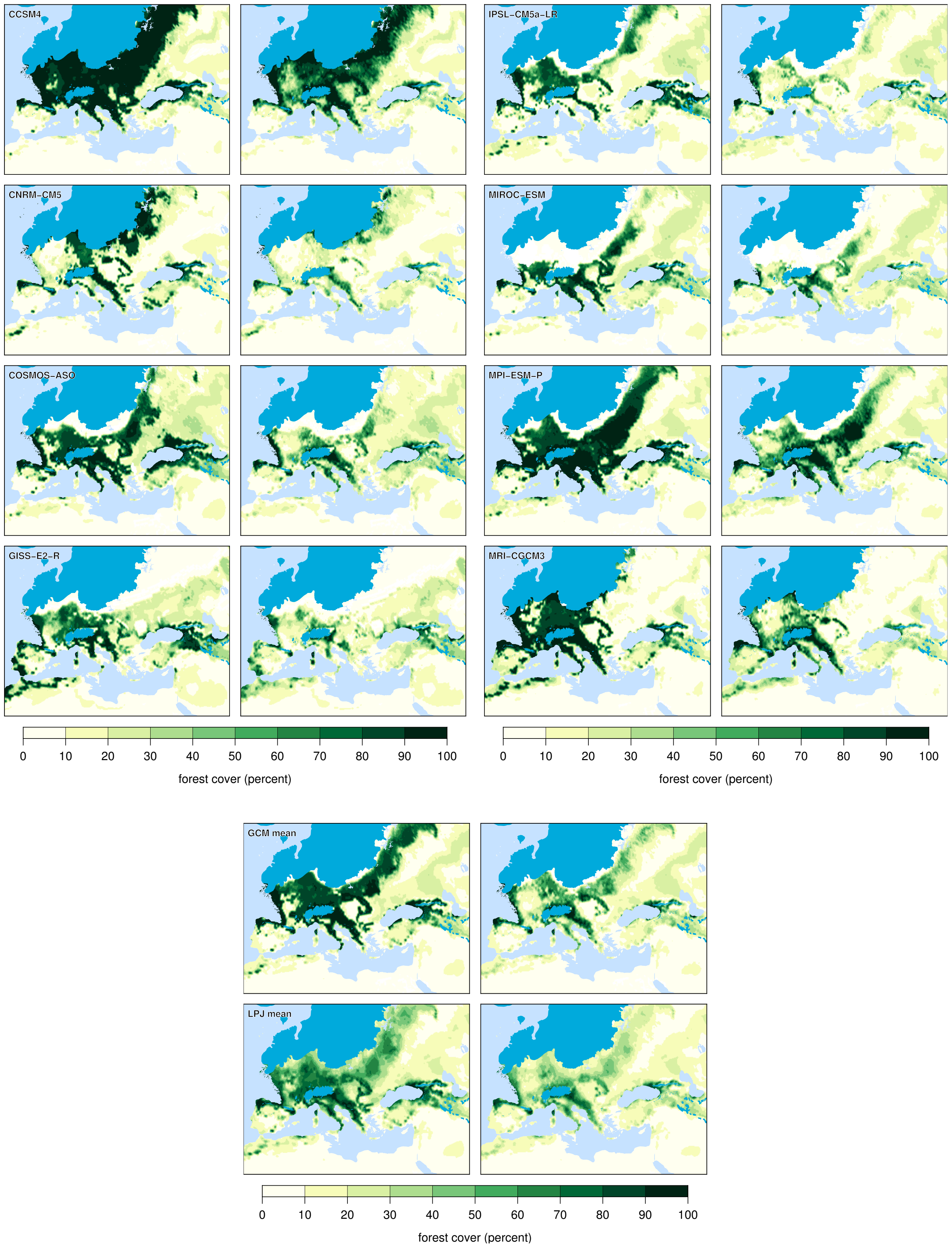

Fig. S1. Tree cover in simulations with and without human burning. Tree cover fraction simulated by LPJ-LMfire using each of the eight GCM LGM climate simulations without human burning (**left panels**) and with human burning (**right panels**). LPJ-LMfire results are also shown for a simulation using the multi-model ensemble mean climate (**GCM mean**), and the mean of all of the individual LPJ-LMfire simulations (**LPJ mean**).
