# Supplementary material for: Large Scale Anthropogenic Reduction of Forest Cover in Last Glacial Maximum Europe
Source: PLoS One. 2016 Nov 30;11(11):e0166726. doi: 10.1371/journal.pone.0166726 (PMC5130213; doi:10.1371/journal.pone.0166726)
Supplement: S2 Fig — The reduction in tree cover between a simulation with and without human burning (left panels) and the associated simulated forager population density (right panels). Results are shown for LPJ-LMfire runs driven by each of the eight GCM climate simulations, and the multi-model ensemble means. (PDF) [file pone.0166726.s002.pdf]

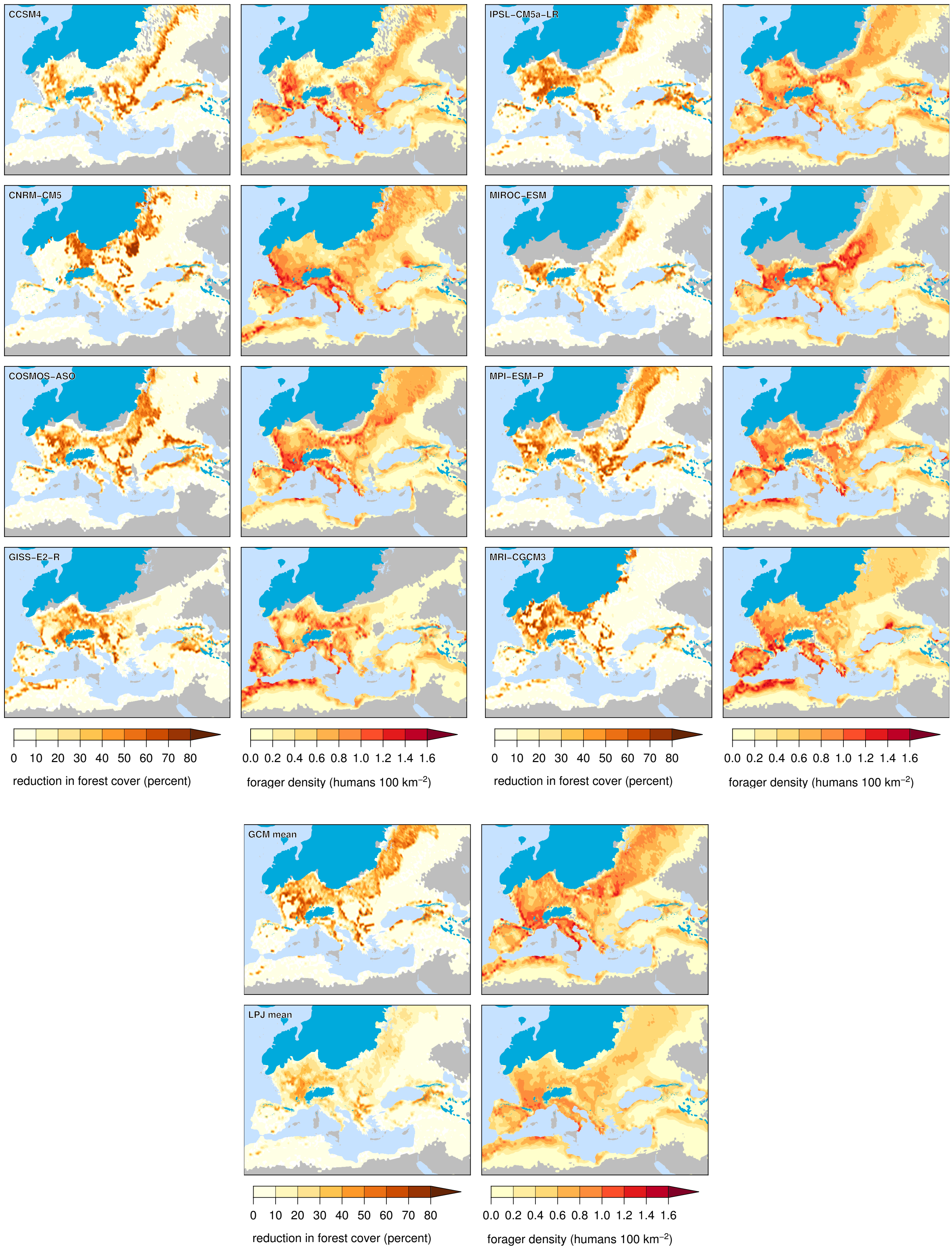


**Fig. S2. Simulated forager population density and associated reduction in tree cover**. The reduction in tree cover between a simulation with and without human burning (**left panels**) and the associated simulated forager population density (**right panels**). Results are shown for LPJ-LMfire runs driven by each of the eight GCM climate simulations, and the multi-model ensemble means.
