# Supplementary material for: Large Scale Anthropogenic Reduction of Forest Cover in Last Glacial Maximum Europe
Source: PLoS One. 2016 Nov 30;11(11):e0166726. doi: 10.1371/journal.pone.0166726 (PMC5130213; doi:10.1371/journal.pone.0166726)
Supplement: S3 Fig — Difference in mean annual burned area fraction for each of the individual GCM LGM climate scenarios compared to a PIH simulation. Left panels, scenarios without human burning at LGM, right panels, with human burning. (PDF) [file pone.0166726.s003.pdf]

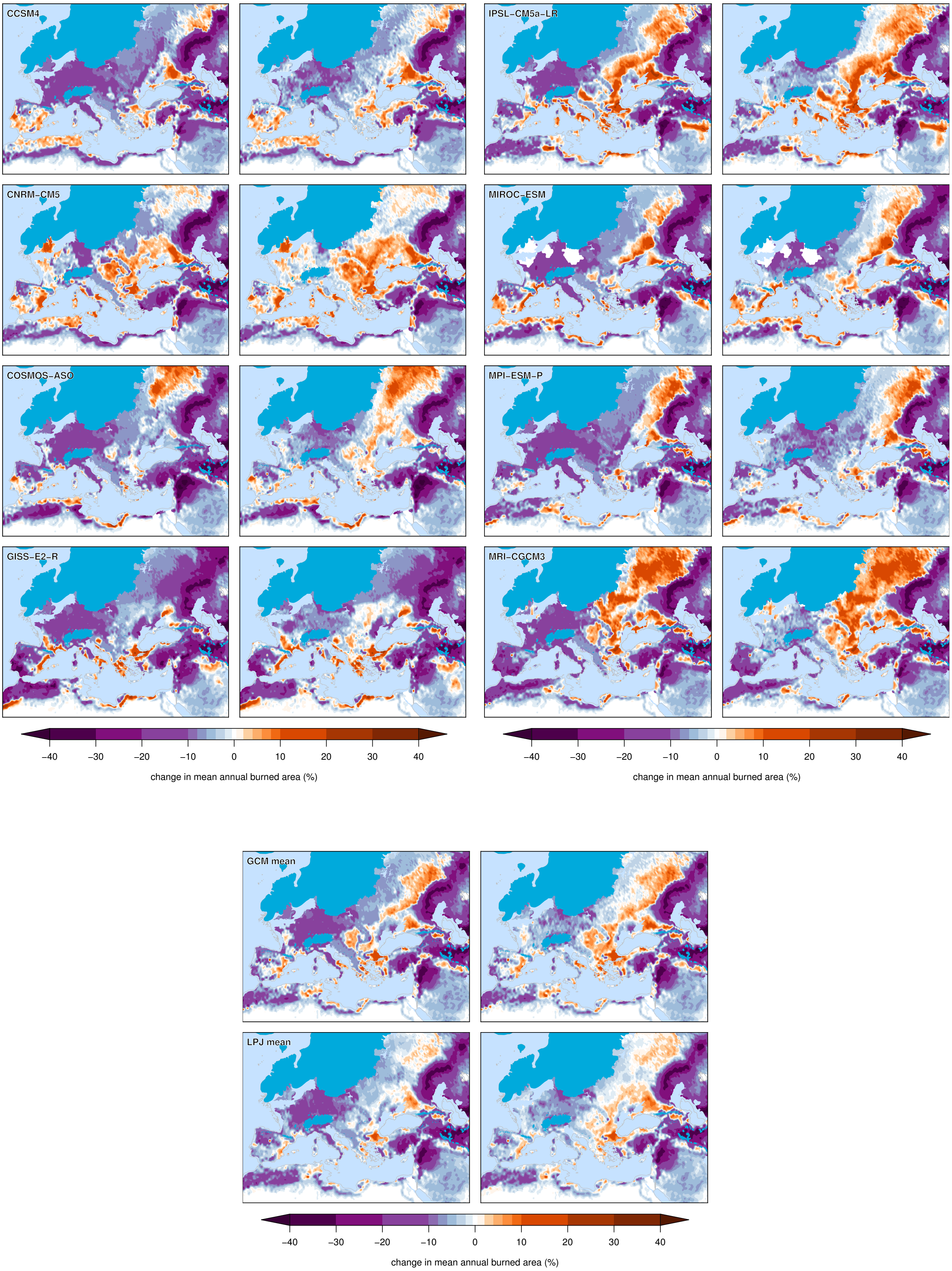

Fig. S3. Change in burned area fraction between LGM and the Preindustrial Holocene (PIH). Difference in mean annual burned area fraction for each of the individual GCM LGM climate scenarios compared to a PIH simulation. **Left panels**, scenarios without human burning at LGM, **right panels**, with human burning.
