# Supplementary material for: Large Scale Anthropogenic Reduction of Forest Cover in Last Glacial Maximum Europe
Source: PLoS One. 2016 Nov 30;11(11):e0166726. doi: 10.1371/journal.pone.0166726 (PMC5130213; doi:10.1371/journal.pone.0166726)
Supplement: S1 Table — Values in parentheses are the 1-σ temporal variability of the individual GCM scenarios, and the 1-σ variability across scenarios for the LPJ mean. (PDF) [file pone.0166726.s004.pdf]

S1 Table. 150-year mean burned area for the map area covered by Fig. 1 for the eight different GCM climate input scenarios, and one scenario using the mean climatology of the 8 different scenarios. Values in parentheses are the 1-σ temporal variability of the individual GCM scenarios, and the 1-σ variability across scenarios for the LPJ mean.

	Total burned area (10^6 km^2)		
GCM scenario	Without humans	With humans	Increase (%)
CCSM4	1.05 (0.10)	1.25 (0.14)	18.8
CNRM-CM5	1.28 (0.14)	1.47 (0.18)	15.4
COSMOS-ASO	0.94 (0.11)	1.20 (0.19)	27.8
GISS-E2-R	0.79 (0.10)	1.01 (0.16)	26.8
IPSL-CM5a-LR	1.32 (0.12)	1.55 (0.17)	17.8
MIROC-ESM	1.00 (0.11)	1.18 (0.19)	18.2
MPI-CGCM3	1.23 (0.14)	1.44 (0.17)	17.8
MPI-ESM-P	1.01 (0.12)	1.23 (0.19)	22.0
**LPJ mean**	**1.08 (0.18)**	**1.29 (0.18)**	**20.6 (4.5)**
GCM mean climate	*1.12 (0.13)*	*1.36 (0.18)*	*20.6*
