# Supplementary material for: Large Scale Anthropogenic Reduction of Forest Cover in Last Glacial Maximum Europe
Source: PLoS One. 2016 Nov 30;11(11):e0166726. doi: 10.1371/journal.pone.0166726 (PMC5130213; doi:10.1371/journal.pone.0166726)
Supplement: S2 Table — Values in parentheses are the 1-σ temporal variability of the individual GCM scenarios, and the 1-σ variability across scenarios for the LPJ mean. (PDF) [file pone.0166726.s005.pdf]

S2 Table. 150-year mean tree cover for the map area covered by Fig. 2 for the eight different GCM climate input scenarios, and one scenario using the mean climatology of the 8 different scenarios. Values in parentheses are the 1-σ temporal variability of the individual GCM scenarios, and the 1-σ variability across scenarios for the LPJ mean.

	Tree cover (10^6 km^2)		
GCM scenario	Without humans	With humans	Decrease (%)
CCSM4	4.93 (0.11)	4.07 (0.09)	17.3
CNRM-CM5	2.78 (0.11)	1.81 (0.07)	35.1
COSMOS-ASO	3.92 (0.09)	2.65 (0.06)	32.4
GISS-E2-R	3.13 (0.10)	2.18 (0.06)	30.4
IPSL-CM5a	3.06 (0.07)	1.94 (0.05)	36.6
MIROC-ESM	2.59 (0.09)	1.88 (0.05)	27.3
MPI-CGCM3	3.40 (0.09)	2.47 (0.05)	27.2
MPI-ESM-P	4.44 (0.08)	3.25 (0.07)	26.8
**LPJ mean**	**3.53 (0.83)**	**2.53 (0.97)**	**29.1 (6.0)**
GCM mean climate	*3.90 (0.10)*	*2.62 (0.06)*	*32.5*
