# Supplementary material for: Large Scale Anthropogenic Reduction of Forest Cover in Last Glacial Maximum Europe
Source: PLoS One. 2016 Nov 30;11(11):e0166726. doi: 10.1371/journal.pone.0166726 (PMC5130213; doi:10.1371/journal.pone.0166726)
Supplement: S3 Table — (A) Complete metadata for entities contributed to the European Pollen Database (EPD) and Pangaea data library may be found at their respective web sites (EPD, Pangaea). (B) Legend for the dating code column in S3A Table. (PDF) [file pone.0166726.s006.pdf]

Table S3A. List of samples used to create the pollen-based reconstruction of tree cover at LGM presented in Fig. 1. Complete metadata for entities contributed to the European Pollen Database (EPD) and Pangaea data library may be found at their respective web sites (EPD, Pangaea). The dating code is explained in table S4.

Site	Source	Site Name	SiteType	Longitude (deg E)	Latitude (deg N)	Elevation (m)	Age of Sample/s (cal. BP)	Dating code	Ref. No. (when not in database)
1	EPD (E#1472)	MD95-2039	Marine Core	-10.35	40.58	-3'381	21'067	1C	
2	Digitised	Carn Morval	Lake	-6.31	49.93	5	20'668	2C	(1)
3	Digitised	Las Devotas	Colluvium	-5.80	42.58	710	22'820	1D	(2)
4	Digitised	Gorham Cave	Cave	-5.30	36.02	0	21'000	1D	(3)
5	Author	Dozmary Pool	Lake	-4.54	50.53	265	21'707	1C	(4)
6	Digitised	Cova di Carihuela	Cave	-3.43	37.45	1'020	19'010	1D	(5)
7	Digitised	Padul	Peat Bog	-3.07	37.00	785	22'056	1C	(6)
8	EPD (E#539)	Quintanar de la Sierra	Lake	-3.02	42.03	1'470	20'446	3C	
9	EPD (E#574)	San Rafael	Peat Bog	-2.60	36.77	0	20'295	1D	
10	Digitised	Siles	Lake	-2.30	38.24	1'320	20'345	1D	(7)
11	Digitised	Torrecilla de Valmadrid	Colluvium	-0.90	41.45	570	20'456	1D	(8)
12	EPD (E#469)	Navarrés	Peat bog	-0.68	39.10	225	19'104	1D	
13	EPD (E#470)	Navarrés	Peat bog	-0.68	39.10	225	19'142	1D	
14	Digitised	El Portalet (peatbog)	Peat Bog	-0.40	42.80	1'802	21'000	4C	(9)
15	Digitised	Tramacastilla paleolake	Lake	-0.40	42.73	1'640	20'600	1D	(2)
16	Digitised	Formigal paleolake	Lake	-0.40	42.79	1'585	20'120	1D	(2)
17	Digitised	Tourbiere de l'Estarres	Lake	-0.38	43.09	356	21'000	1C	(10)
18	Digitised	Cova de les Malladetes	Cave	-0.32	39.06	20	19'686	1D	(11)
19	EPD (E#972)	Saint-Ursin	Peat Bog	-0.25	48.52	234	21'945	6C	
20	Digitised	Biscaye	Lake	-0.08	43.03	409	20'163	6C	(12)
21	Digitised	Lourdes	Lake	-0.08	43.03	430	20'730	1C	(12)
22	Digitised	Lake Estanya	Lake	0.53	42.03	670	19'500	1D	(13)
23	Digitised	Freychinede	Lake	1.43	42.78	1'350	21'000	2C	(14)
24	EPD (E#931)	Banyoles	Lake	2.75	42.13	173	21'008	1D	
25	EPD (E#1293)	Lac du Bouchet	Lake	3.78	44.92	1'200	21'084	1C	
26	EPD (E#1474)	MD99-2348 (103)	Marine core	3.84	42.69	-296	20'657	1C	
27	EPD (E#381)	Le Grand Lemps	Peat bog	5.42	45.47	500	19'824	3C	
28	Digitised	La Grotte Walou	Cave	5.54	50.59	252	21'200	1D	(15)
29	EPD (E#920)	Peuil Peat Bog	Peat bog	5.64	45.13	970	19'690	6C	
30	EPD (E#933)	Correo	Peat bog	5.98	44.51	1'090	20'810	1D	
31	EPD (E#935)	La Grande Pile	Peat bog	6.50	47.73	330	20'859	1C	
32	Digitised	Laghi dell'Orgials	Lake	7.04	44.04	2'240	21'000	6C	(16)
33	EPD (E#394)	Lobsigensee	Lake	7.30	47.03	514	19'356	4C	
34	EPD (E#1452)	Lago Piccolo di Avigliana	Lake	7.39	45.23	356	19'361	5C	
35	EPD (E#1125)	Chutti Boltigen	Peat bog	7.39	46.63	925	20'938	6C	
36	EPD (E#63)	Amsoldingersee	Lake	7.58	46.72	641	20'294	6C	
37	Digitised	Pilsensee	Lake	11.19	48.03	534	21'000	1D	(17)
38	EPD (E#1208)	Lanser Moor	Peat Bog	11.42	47.24	840	19'511	4C	
39	Digitised	Orgiano	Peat Bog	11.43	45.29	19	20'531	1C	(18)
40	Digitised	Lago della Costa	Lake	11.74	45.27	7	21'000	1C	(19)
41	EPD (E#916)	Lagaccione	Lake	11.85	42.57	355	20'925	1C	
42	EPD (E#929)	Lago Vico	Lake	12.17	42.32	510	21'257	1C	

Site	Source	Site Name	SiteType	Longitude (deg E)	Latitude (deg N)	Elevation (m)	Age of Sample/s (cal. BP)	Dating code	Ref. No. (when not in database)
43	EPD (E#1186)	Egelsee	Lake	12.17	47.61	549	19'210	6C	
44	Digitised	Stracciacappa	Lake	12.32	42.13	220	21'000	3D	(20)
45	Digitised	Lago di Monterosi	Lake	12.43	42.22	237	21'616	1D	(21)
46	Digitised	Travesio	Lake	12.87	46.20	220	22'483	1D	(22)
47	Digitised	Azzano Decimo	Outwash plain	12.90	45.87	10	21'637	1D	(23)
48	EPD (E#179)	Colfiorito	Peat bog	12.93	43.03	752	19'210	6C	
49	Digitised	Rio Ponte	Lake	12.98	46.18	280	22'909	1D	(22)
50	Digitised	Rio Doidis	Lake	13.19	46.12	152	22'691	1D	(22)
51	Digitised	Billerio	Lake	13.21	46.22	300	21'872	1D	(22)
52	Digitised	Castiglione	Lake	13.25	42.48	44	21'000	1C	(24)
53	Digitised	Kersdorf-Briesen	Lake	14.27	52.33	44	21'183	1D	(25)
54	EPD (E#932)	Lago Grande di Monticchio	Lake	15.60	40.94	1'326	19'703	1C	
55	EPD (E#1397)	Nagy-Mohos	Peat bog	20.44	48.33	270	21'044	1C	
56	Digitised	Safarka	Peat bog	20.58	48.88	600	21'912	1D	(26)
57	Digitised	Feher Lake	Lake	20.65	46.45	86	21'249	1C	(27)
58	Pangaea	Lake Ohrid	Lake	20.76	40.94	693	20'845	6C	
59	Digitised	Lake Prespa	Lake	20.98	40.96	849	21'000	6D	(28)
60	EPD (E#976)	Lake Xinias	Lake	22.27	39.05	500	20'650	4C	
61	EPD (E#18)	Avrig	Peat bog	24.38	45.72	400	19'148	4C	
62	Digitised	Mickunai	Lake	25.53	54.72	143	21'000	1D	(29)
63	Digitised	Lake Sfanta Anna (Saint Anne)	Lake	25.89	46.13	946	20'955	1D	(30)
64	Digitised	Lesvos ML01 Megali Limni	Lake	26.30	39.10	323	22'906	1D	(31)
65	Digitised	Atolov Straldzha	Lake	26.77	42.63	138	21'000	5D	(32)
66	EPD (E#319)	Kararmik Batakligi	Peat bog	30.80	38.43	1'000	20'722	5D	
67	EPD (E#977)	Yeniçaga Gölü	Peat bog	32.03	40.78	987	19'046	6C	
68	Pangaea	M72/5_628-1	Marine core	36.62	42.10	-418	21'257	1C	
69	Digitised	Dziguta_Core 1	Peat bog	41.07	42.99	35	21'000	2C	(33)
70	Pangaea	Lake Van LG	Lake	42.67	38.67	1'649	21'019	1C	
71	EPD (E#714)	Lake Zeribar	Lake	46.12	35.53	1'286	20'794	3C	

Table S3B. Legend for the dating code column in Table S3A

Code	Description
1C	Bracketing dates within 2000 yr interval about the time being assessed
2C	Bracketing dates, one within 2000 yr and the second within 4000 yr of the time being assessed
3C	Bracketing dates within 4000 yr interval about the time being assessed
4C	Bracketing dates, one being within 4000 yr and the second being within 6000 yr of the time being assessed
5C	Bracketing dates within 6000 yr interval about the time being assessed
6C	Bracketing dates, one within 6000 yr and the second within 8000 yr of the time being assessed
7C	Poorly dated
1D	Date within 250 yr of the time being assessed
2D	Date within 500 yr of the time being assessed
3D	Date within 750 yr of the time being assessed
4D	Date within 1000 yr of the time being assessed
5D	Date within 1500 yr of the time being assessed
6D	Date within 2000 yr of the time being assessed
7D	Poorly dated

References for Table S3A

1. Scourse JD (1991) Late Pleistocene Stratigraphy and Paleobotany of the Isles of Scilly. *Philos T Roy Soc B* 334(1271):405-448.
2. Gonzalez-Samperiz P*, et al.* (2005) Glacial and Lateglacial vegetation in northeastern Spain: New data and a review. *Quaternary International* 140:4-20.
3. Carrion JS*, et al.* (2008) A coastal reservoir of biodiversity for Upper Pleistocene human populations: palaeoecological investigations in Gorham's Cave (Gibraltar) in the context of the Iberian Peninsula. *Quaternary Sci Rev* 27(23-24):2118-2135.
4. Kelly A, Charman DJ, & Newnham RM (2010) A Last Glacial Maximum pollen record from Bodmin Moor showing a possible cryptic northern refugium in southwest England. *J Quaternary Sci* 25(3):296-308.
5. Fernandez S*, et al.* (2007) The Holocene and Upper Pleistocene pollen sequence of Carihuela Cave, southern Spain. *Geobios* 40(1):75-90.
6. Pons A & Reille M (1988) The Holocene-Pleistocene and Upper-Pleistocene Pollen Record from Padul (Granada, Spain) - a New Study. *Palaeogeogr Palaeocl* 66(3-4):243-263.
7. Carrion JS (2002) Patterns and processes of Late Quaternary environmental change in a montane region of southwestern Europe. *Quaternary Sci Rev* 21(18-19):2047-2066.
8. Valero-Garces BL*, et al.* (2004) Paleohydrological fluctuations and steppe vegetation during the last glacial maximum in the central Ebro valley (NE Spain). *Quaternary International* 122:43-55.
9. Gonzalez-Samperiz P*, et al.* (2006) Climate variability in the Spanish Pyrenees during the last 30,000 yr revealed by the El Portalet sequence. *Quaternary Research* 66(1):38-52.
10. Jalut G, Andrieu V, Delibrias G, Fontugne M, & Pages P (1988) Palaeoenvironment of the valley of Ossau (Western French Pyrénées) during the last 27,000 years. *Pollen et Spores* 30(3-4):357-394.
11. Ollivier MD (1988) *Palinología y Paleoambiente: Nuevos datos españoles. Referencias* (Museu de Prehistòria de Valencia, Valencia) p 176.
12. Reille M & Andrieu V (1995) The Late Pleistocene and Holocene in the Lourdes Basin, Western Pyrenees, France - New Pollen Analytical and Chronological Data. *Veg Hist Archaeobot* 4(1):1-21.
13. Vegas-Vilarrubia T*, et al.* (2013) Diatom and vegetation responses to Late Glacial and Early Holocene climate changes at Lake Estanya (Southern Pyrenees, NE Spain). *Palaeogeogr Palaeocl* 392:335-349.
14. Jalut G*, et al.* (1992) Glacial to Interglacial Vegetation Changes in the Northern and Southern Pyrenees - Deglaciation, Vegetation Cover and Chronology. *Quaternary Sci Rev* 11(4):449-480.
15. Damblon F, Court-Picon M, & Pirson S (2011) L'enregistrement palynologique de la séquence pléistocène et holocène de la grotte Walou (Trooz, Province de Liège, Belgique). *Recherches à la grotte Walou à Trooz (province de Liège, Belgique),* Etudes et Documents, Archaeologie, eds Draily C, Pirson S, Juvigné E, Renson V, Toussaint M, & Turmes M (Société Wallonne de Palethnologie, Liège), Vol 2, pp 82-129.
16. Ortu E, David F, & Caramiello R (2005) Effect of local parameters on the development of vegetation in the Valley of St. Anna di Vinadio (Maritime Alps, Italy). *Ecoscience* 12(1):122-135.
17. Küster H (1995) *Postglaziale Vegetationsgeschichte Südbayerns: Geobotanische Studien zur prähistorischen Landschaftskunde* (Akademie Verlag, Berlin) p 372.
18. Paganelli A (1996) Evolution of vegetation and climate in the Veneto-Po plain during the late-glacial and the Early Holocene using pollen stratigraphic data. *Il Quaternario* 9(2):581-590.
19. Kaltenrieder P*, et al.* (2009) Environmental and climatic conditions at a potential Glacial refugial site of tree species near the Southern Alpine glaciers. New insights from multiproxy sedimentary studies at Lago della Costa (Euganean Hills, Northeastern Italy). *Quaternary Sci Rev* 28(25-26):2647-2662.
20. Giardini M (2007) Late Quaternary vegetation history at Stracciacappa (Rome, central Italy). *Veg Hist Archaeobot* 16(4):301-316.
21. Bonatti E (1970) Pollen Sequence in the Lake Sediments. *Ianula: An Account of the History and Development of the Lago di Monterosi, Latium, Italy,* Transactions of the American Philosophical Society, eds Hutchinson GE, Bonatti E, Cowgill UM, Goulden CE, Leventhal EA, Mallett ME, Margaritora F, Patrick R, Racek A, Roback SA, *et al.* (The American Philosophical Society, Philadelphia), Vol 60, pp 26-31.
22. Monegato G*, et al.* (2007) Evidence of a two-fold glacial advance during the last glacial maximum in the Tagliamento end moraine system (eastern Alps). *Quaternary Research* 68(2):284-302.
23. Pini R, Ravazzi C, & Donegana M (2009) Pollen stratigraphy, vegetation and climate history of the last 215 ka in the Azzano Decimo core (plain of Friuli, north-eastern Italy). *Quaternary Sci Rev* 28(13-14):1268-1290.

24. Alessio M, *et al.* (1986) ^{14}C dating, geochemical features, faunistic and pollen analyses of the uppermost 10 m core from Valle di Castiglione (Rome, Italy). *Geologica Romana* 25:287-308.
25. Strahl J (2005) Zur Pollenstratigraphie des Weichselspätglazials von Berlin-Brandenburg. *Brandenburger geowissenschaftliche Beiträge* 12(1/2):87-112.
26. Jankovská V (2008) Vegetation cover in West Carpathians during the Last Glacial Period - analogy of present day Siberian forest-tundra and taiga. in *Palynology: stratigraphy and geoecology. Collection of the Scientific Works of XII All-Russian Palynological conference (29 of September – 4 October, 2008* (Saint-Petersburg).
27. Magyari EOK, *et al.* (2014) Late Pleniglacial vegetation in eastern-central Europe: are there modern analogues in Siberia? *Quaternary Sci Rev* 95:60-79.
28. Panagiotopoulos K, Bohm A, Leng MJ, Wagner B, & Schabitz F (2014) Climate variability over the last 92 ka in SW Balkans from analysis of sediments from Lake Prespa. *Clim Past* 10(2):643-660.
29. Satkūnas J & Grigienė A (2012) Eemian-Weichselian palaeoenvironmental record from the Mickūnai glacial depression (Eastern Lithuania). *Geologija* 54(2):35-51.
30. Magyari EK, *et al.* (2014) Vegetation and environmental responses to climate forcing during the Last Glacial Maximum and deglaciation in the East Carpathians: attenuated response to maximum cooling and increased biomass burning. *Quaternary Sci Rev* 106:278-298.
31. Margari V, Gibbard PL, Bryant CL, & Tzedakis PC (2009) Character of vegetational and environmental changes in southern Europe during the last glacial period; evidence from Lesvos Island, Greece. *Quaternary Sci Rev* 28(13-14):1317-1339.
32. Connor SE, *et al.* (2013) Environmental conditions in the SE Balkans since the Last Glacial Maximum and their influence on the spread of agriculture into Europe. *Quaternary Sci Rev* 68:200-215.
33. Arslanov KA, Dolukhanov PM, & Gei NA (2007) Climate, Black Sea levels and human settlements in Caucasus Littoral 50,000-9000 BP. *Quaternary International* 167:121-127.
